# SAYCam: A Large, Longitudinal Audiovisual Dataset Recorded From the Infant’s Perspective

**DOI:** 10.1162/opmi_a_00039

**Published:** 2021-05-26

**Authors:** Jessica Sullivan, Michelle Mei, Andrew Perfors, Erica Wojcik, Michael C. Frank

**Affiliations:** Skidmore College; Skidmore College; University of Melbourne; Skidmore College; Stanford University

**Keywords:** headcam, first-person video, child development

## Abstract

We introduce a new resource: the SAYCam corpus. Infants aged 6–32 months wore a head-mounted camera for approximately 2 hr per week, over the course of approximately two-and-a-half years. The result is a large, naturalistic, longitudinal dataset of infant- and child-perspective videos. Over 200,000 words of naturalistic speech have already been transcribed. Similarly, the dataset is searchable using a number of criteria (e.g., age of participant, location, setting, objects present). The resulting dataset will be of broad use to psychologists, linguists, and computer scientists.

## INTRODUCTION

From the roots of children’s language learning to their experiences with objects and faces, naturalistic data about children’s home environment provides an important constraint on theories of development (Fausey et al., 2016; MacWhinney, 2000; Oller et al., 2010). By analyzing the information available to children, researchers can make arguments about the nature of children’s innate endowment and their learning mechanisms (Brown, 1973; Rozin, 2001). Further, children’s learning environments are an important source of individual variation between children (Fernald et al., 2012; Hart & Risley, 1992; Sperry et al., 2019); hence, characterizing these environments is an important step in developing interventions to enhance or alter children’s learning outcomes.

When datasets—for example, the transcripts stored in the Child Language Data Exchange System (MacWhinney, 2000)—are shared openly, they form a resource for the validation of theories and the exploration of new questions (Sanchez et al., 2019). Further, while tabular and transcript data were initially the most prevalent formats for data sharing, advances in storage and computation have made it increasingly easy to share video data and associated metadata. For developmentalists, one major advance is the use of Databrary, a system for sharing developmental video data that allows fine-grained access control and storage of tabular metadata (Gilmore & Adolph, 2017). The advent of this system allows for easy sharing of video related to children’s visual experience. Such videos are an important resource for understanding children’s perceptual, conceptual, linguistic, and social development. Because of their richness, such videos can be reused across many studies, making the creation of open video datasets a high-value enterprise.

One especially promising method for characterizing children’s visual experience is the head-mounted camera (Aslin, 2012; Franchak et al., 2011), a lightweight camera or eye-tracker that is worn by the child, often mounted on a hat or harness system. A “headcam” allows access to information from the perspective of the child–albeit with some differences in view angle, resolution, and orienting latency (Pusiol et al., 2014; Smith et al., 2015). Headcam data have been used in recent years to understand a variety of questions about children’s visual input, including the prevalence of social signals (Fausey et al., 2016), how children’s bodies and hands shape their attention (Bambach et al., 2016), how children interact with adults (Yu & Smith, 2013), which objects are in their visual field (Cicchino et al., 2011), and how their motor development shapes their visual input (Kretch et al., 2014; Sanchez et al., 2019). These datasets also let researchers access some of the “source data” for children’s generalizations about object identity or early word learning (Clerkin et al., 2017). Data from headcams are also an important dataset for studies of unsupervised learning in computer vision (Bambach et al., 2016). Yet there are relatively few headcam datasets available publicly for reuse, and those that are almost exclusively report cross-sectional, in-lab data (Franchak et al., 2018; Sanchez et al., 2018), or sample from one developmental time point (Bergelson, 2017).

The current project attempts to fill this gap by describing a new, openly accessible dataset of more than 415 hours of naturalistic, longitudinal recordings from three children. The SAYCam corpus contains longitudinal videos of approximately two hr per week for three children spanning from approximately six months to two and a half years of age. The data include unstructured interactions in a variety of contexts, both indoor and outdoor, as well as a variety of individuals and animals. The data also include structured annotations of context together with full transcripts for a subsample (described below), and are accompanied by monthly parent reports on vocabulary and developmental status. Together, these data present the densest look into the visual experience of individual children currently available.

## METHOD

### Participants

Three families participated in recording head-camera footage (see Table 1). Two families lived in the United States during recording, while one family lived in Australia. All three families spoke English exclusively. In all three families, the mother was a psychologist. All three children had no siblings during the recording window. This research was approved by the institutional review boards at Stanford University and Skidmore College. All individuals whose faces appear in the dataset provided verbal assent, and, when possible, written consent. All videos have been screened for ethical content.

**Table 1.** Participant information

**Participant**	**Location**	**Age at first recording (months)**	**Age at last recording (months)**
Sam (S)	Adelaide, Australia	6 m.	30 m.
Alice (A)	San Diego, California, USA and Saratoga Springs, New York, USA	8 m.	31 m.
Asa (Y)	Saratoga Springs, New York, USA	7 m.	24 m.

Sam is the child of the family who lived in Australia. He wore the headcam from 6 months to 30 months of age. The family owned two cats and lived in a semirural neighborhood approximately 20 miles from the capital city of Adelaide. Sam was diagnosed with autism spectrum disorder at age 3; as of this writing (at age 7), Sam is fully mainstreamed, has friends, and does not require any special support.

Alice is the child of a family who lived in the United States. She wore the headcam from 8 months to 31 months of age. The family also owned two cats throughout the recording period. For the first half of the recordings, the family lived on a rural farm and kept other animals on or near their property, including chickens; the family frequently visited a major city (San Diego). For the second half of the recordings, the family lived in the suburbs in New York State.

Asa is the child of a family who lived in the United States. He wore the headcam from 7 months to 24 months of age. Data collection for this child terminated prematurely due to the onset of the COVID-19 pandemic and the birth of a younger sibling. The family owns no pets, and lives in the suburbs in New York State.

### Materials

Parents completed the MacArthur-Bates Communicative Development Inventories (MCDI) inventory each month (Fenson et al., 2007), as well as the Ages and Stages Questionnaire (Squires et al., 1997) during the time periods required by the instrument. These materials are included in the corpus, and can be used to identify the age at which each participant acquired particular words, particular motor capacities, and particular social and cognitive capacities. These data may be useful for characterizing the children (e.g., for their representativeness), but also for identifying particular videos (described below) recorded immediately before or after particular developmental milestones (e.g., for finding videos from before a child learned a particular word, or became able to stand independently).

**Figure 1.**
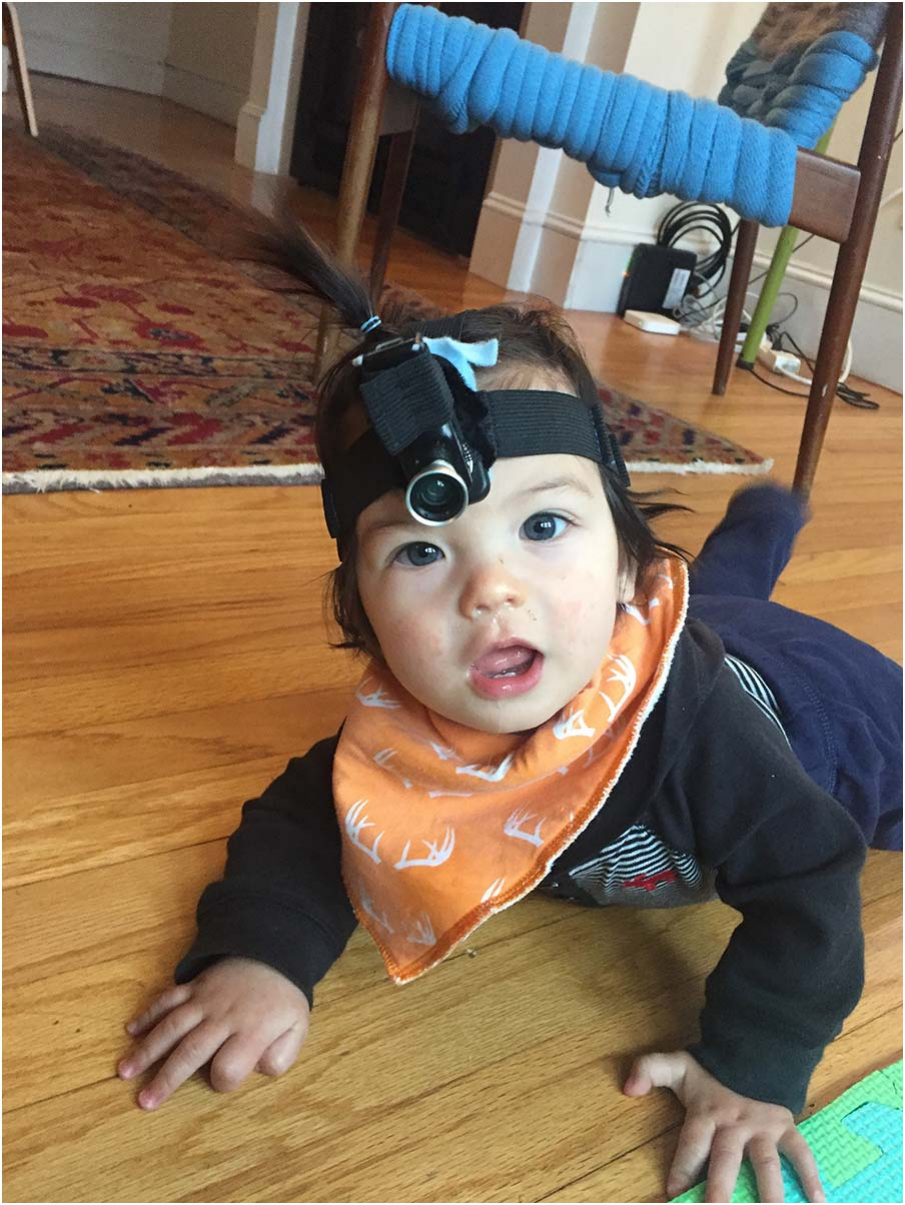
Participant (7 months old) wearing Veho camera with fish eye lens.

Videos were recorded using a Veho head-mounted camera on a custom mounting headband (Figure 1). Each Veho MUVI Pro micro DV camcorder camera was equipped with a magnetic mount that allowed the attachment of a wide-angle fisheye lens to broaden the MUVI Pro’s native 47- by 36-degree viewing angle to 109 by 70 degrees. We selected the Veho MUVI camera after testing several headcams in-lab, and determining it provided the biggest vertical visual angle and had physical dimensions that allowed for comfortable mounting near the center of the forehead (giving a perspective that would be most similar to the child’s; see Long et al., 2020, for a comparison of headcams). According to the specs provided at purchase, the camera captures video at a resolution of 480 p, and at up to 30 frames per s, although in practice, the frame rate sometimes dipped to approximately 20 frames per s. Audio quality from the cameras was highly variable, and in some cases, the camera’s default audio recordings are low quality.

On occasions where a child persistently refused to wear the headcam, the camera was used as a normal camera (i.e., placed nearby and carried to new locations if necessary). A spreadsheet detailing which videos are third person accompanies our data on Databrary (https://nyu.databrary.org/volume/564/slot/47832/edit?asset=273993).

## PROCEDURE

Videos were recorded naturalistically, in and around the homes, cars, neighborhoods, and workplaces where the child spent time. The intention was to record videos twice per week; once at a fixed time and once at a randomly chosen time. Given the practical constraints of a multiyear project like this one, at times there were deviations from the planned procedure. For example, in the Alice dataset, there were some weeks where both recording times were determined pseudo-randomly because the fixed recording time was not feasible. Similarly, in the Sam dataset, scheduling constraints occasionally meant that there was less variation in the timing of recordings than one might otherwise expect. In the Asa dataset, the pseudo-random time often occurred at a fixed time due to feasibility issues (e.g., attendance at day care), and occasional weeks were skipped due to travel or other logistical barriers; additionally, most recordings for the Asa dataset are sampled from between 7 a.m. and 10 a.m. Technical issues, such as memory card or camera failure, although infrequent, resulted in some skipped recording sessions.

When recording, we obtained written consent or verbal assent (in accordance with institutional review board) for participants whose face and voice were featured in the videos. Videos for which we were unable to secure assent/consent for people who were potentially identifiable are omitted from the dataset.

Each recording session lasted until the battery on the Veho camera failed, or once 90 min had elapsed. In most cases, this resulted in recording sessions that lasted approximately 60 to 80 min. Each recording session typically resulted in multiple video files, with a maximum single-file duration of 30 min.

### Coding

One goal of our coding efforts was to tag each video with its contents, so that researchers could search for content relevant to their research interests. To do this, videos were skimmed at an effective 10x-speed playback, and visually assessed for locations and location changes. Coding occurred by playing each video in VLC (VideoLAN Client), and coding using custom spreadsheets with macros. Coders were highly trained and deeply familiar with the dataset.

The following pieces of information were coded for each video. First, the coder briefly described the contents of the video (e.g., “Picked grass to play with during walk”; “On floor looking at mirror with mom”; “watching parents in kitchen”). The coder also recorded the location (e.g., hallway, bedroom, kitchen, outdoors, car, …), the activity or activities (e.g., being held, walking, cooking, drinking, eating, listening to music, …), the individual(s) present (e.g., child, dad, mom, grandparent, animal, …), the body parts visible (e.g., arms/hands, legs/feet, head/face), and the most salient objects (e.g., flora, drink, tool, food, bed, book, …). See Table 2 for a full list of coding classifications. Criteria for each coding decision are outlined in our Read Me document on Databrary (https://nyu.databrary.org/volume/564/slot/47832/-?asset=254904). The coder also noted any potential ethical concerns for further screening, and any additional notes.

**Table 2.** Locations, Activities, Living Things, and Objects that are searchable in our corpus

**Locations**	**Activity**	**Living Things**	**Objects**	**Body Parts**
Bathroom	Being Held	Bird(s)	Appliance	Present/Absent
Bedroom	Cleaning	Cat (with specific cat)	Bag	Head/Face
California Room	Cooking	Dad	Bed	Body
Car	Coversing	Dog	Book	Arms/Hands
Closet	Crawling	Fish	Building	Legs/Feet
Deck/Porch	Crying	Grandma	Car	Other
Garage	Drawing	Grandpa	Chair	Phone*
Hallway	Drinking	Horse	Clothing	
Kitchen	Eating	Mom	Computer	
Laundry Room	Examining	Other Adult	Container	
Living Room	Exploring	Other Child	Cream	
Off Property	Gardening	Participant	Crib	
Office	Getting Changed/Dressed		Diapers/Wipes/Potty	
Outside On Property	Getting Parental Attention		Doll/Stuffed Toy	
Piano Room	Imitating		Door	
Many Locations	Listening to Music		Drawing	
Stairway	Lying Down		Drawing/Writing Implements	
	Nursing		Drink	
	Overhearing Speech		Flora	
	Painting		Food	
	Playing Music		Improvised Toy	
	Preparing for Outing		Laundry Machine	
	Reading		Linen/Cloths	
	Running		Mirror	
	Sitting		Musical Instrument	
	Standing		Outdoors	
	Taking a Walk		Phone	
	Tidying		Picture	
	Walking		Play Equipment	
	Watching		Puzzle	
			Table	
			Tool	
			Toy	
			Wagon	
			Window	

*Note*. In some cases, individuals were present in the dataset via phone and therefore were not embodied; for those cases, we indicated the individual’s presence via “Phone” in the “Body Parts” section.

Every time there was a significant change in location or in activity, we coded each component of the video anew. For example, if the child moved from the bedroom to the bathroom, we coded all elements of the video (e.g., objects, activity, body parts, individuals) once for the bedroom and once for the bathroom.

The intention for coding videos was to allow other researchers to identify videos of interest, not for analysis of the codes themselves. While such an analysis may be both possible and extremely interesting in the future, we encourage caution at this point for several reasons. First, relative frequencies of activities will not be accurately computed until the entire dataset is coded. Second, while our coding scheme remained stable throughout the majority of coding, there are a few items (e.g., the location “Laundry Room,” the item “cream,” the activity “patting”) that were added to our coding scheme after coding began. Their dates of addition are described in the Read Me file. Finally, some objects, activities, or locations may appear briefly in particular videos, but not be tagged (e.g., if the child is in the bedroom, throws a ball, and briefly looks into the hallway to see if the ball landed there, only “location: bedroom” may be tagged). The decision for whether or not to tag a particular entity within the video was subjective, and was guided by the intention of coding the videos in a way that would be helpful for future researchers to identify videos that would be useful for their particular research projects. For example, in the current dataset, the tag “Object: Clothing” labels 114 videos (i.e., the videos where clothing was a salient feature of the child’s experience), and researchers who are interested in children’s interactions with clothing will find at least one salient child–clothing interaction in each of those videos. However, the absence of the “Object: Clothing” tag for the remaining videos certainly does not imply that everyone was naked.

Researchers interested in contributing to the ongoing coding effort should contact the corresponding author.

### Transcription

One goal of this project was to transcribe all of the utterances in each video. Videos were assigned to college students and transcribed. Interested readers can contribute to the ongoing transcription effort by contacting the corresponding author; our original training materials are provided here: https://nyu.databrary.org/volume/564/slot/47832/-?asset=254904. Transcribers noted the timestamp for the onset of each utterance, the contents of the utterance itself, the speaker, any notable, significant, or contextually important actions, and any objects that were relevant, visually salient, or interacted with. Transcribers were instructed to record any utterances they heard verbatim. A new entry was created every time there was a change in speaker, there was a meaningful shift in conversation, or there was a meaningful pause between speaking. Each entry begins by recording the time in the video that the utterance in question begins.

## RESULTS

There are over 1,800 videos with more than 460 unique recording sessions, yielding well over 500 hr of footage. Currently, over 235,800 words have been transcribed, representing 16.5% of the present dataset; 28.3% of the videos have been coded for location, activity, living things, body parts, and objects and are now searchable.

Researchers can access our dataset, which contains AVI and MP4 videos, PDFs, and Excel sheets representing our data at the Supplementary Materials online, and can search for relevant videos here: https://skidmoreheadcam.shinyapps.io/SAYcam-TagSearch/. For example, a researcher interested in studying the auditory input surrounding infant nursing could visit our search tool, and search for segments containing nursing. At present, this would yield 53 videos; the output table automatically includes a link to the relevant video, and a link to a Google Sheet containing any available transcriptions. The researcher could then choose to analyze those 53 videos, or to further restrict their search (e.g., by age, other individuals present, other activities surrounding the event). Or, let’s say a researcher was interested in a child’s exposure to cats prior to acquiring the word “cat.” The researcher would then access the child’s MCDI vocabulary measures on Databrary in order to identify the month at which the child acquired the word “cat.” They could then use our search tool to search for videos from that child from *before* the child learned the word, and access those videos via the provided Databrary link.

Individuals can apply to access the data using Databrary’s (https://nyu.databrary.org/) standard application process. Researchers will need to demonstrate that they are authorized investigators from their institution, that they completed ethics training, and that their institution has an institutional review board.

## DISCUSSION

Beginning with early diary studies and continuing with video recordings of parents–child interaction, naturalistic corpora have helped researchers both ensure the ecological validity of theories of learning and development and generate new research questions (e.g., Brown, 1973; MacWhinney, 2000). The current dataset is a novel contribution to naturalistic developmental corpora in several ways. First, the use of head-mounted cameras constrains the video recordings to the child’s point of view, allowing researchers to not simply observe the visual stimuli surrounding a child, but also access the visual stimuli that is actually available to a child (see Yurovsky et al., 2013, for an example of how this first-person perspective can lead to novel insights into learning mechanisms). Second, the biweekly recording schedule led to the creation of a dataset with finer grained sampling than in most other longitudinal corpora (see Adolph & Robinson, 2011, for a discussion of the importance of sampling rate). Third, the two-year span of recordings is longer than most longitudinal datasets, allowing for researchers to track development in a way that is not possible for shorter projects. Lastly, the open sharing of the data on Databrary gives researchers (and their students) the rare opportunity to freely and easily access the entire corpus.

We anticipate our data will be a valuable tool for researchers in developmental science, and in particular to those interested in categorization and language learning. One of the most important questions in language acquisition is how children connect words with their meanings. This is a difficult problem for the language learner, because for any amount of evidence about the use of a word, there is an infinite range of meanings that are consistent with that evidence (Quine, 1960). Viewed as a categorization problem, the challenge posed to children is to identify the extension of categories in the world and, simultaneously, to infer which labels map onto those categories. For example, a child must both determine that the word “cat” refers to this particular cat and also that more generally it refers to a concept that includes other cats, but not dogs or horses. This process may depend critically on the distribution of the objects and entities in the child’s environment, the use of object labels in the child’s environment, and the distributional relationship of the overlap between objects and labels—the present dataset uniquely allows us to track children’s naturalistic audio and visual input, and to relate it to the trajectory of their word learning.

In addition, this dataset will be of use for those interested in quantifying children’s early naturalistic environments. Previous work (Yoshida & Smith, 2008) has shown that headcam data is a viable proxy for infants’ eye movements, and that headcam footage is valuable for exploring the composition of a child’s visual field (Aslin, 2009). We have previously used headcam data to investigate how changing motor abilities affect children’s experience (Frank et al., 2013), while others have used headcams to show the changing composition of children’s visual input in early development (Fausey et al., 2016). Our longitudinal, naturalistic, first-person dataset will allow researchers to ask and answer questions about the statistic of dyadic interactions across time: our archive provides researchers with access to data on joint attention, infant gaze, parental dyadic engagement, attachment, in addition to the basic statistics of the infant’s perceptual experience. Indeed, early work using our data already suggests interesting insights can be extracted by considering how social information is modulated by different activity contexts (Long et al., 2020).

Finally, the density of our dataset is extremely important for the use of new neural network algorithms, which tend to be “data hungry.” Already, researchers have explored what learning progress is possible when new unsupervised visual learning algorithms are applied to our dataset (Orhan et al., 2020; Zhuang et al., 2020). In fact, as Orhan et al. (2020) note, even though our dataset is quite large by conventional standards, a child’s visual experience from birth to age two and a half would in fact be two orders of magnitude larger still, and the machine learning literature suggests that such an increase in data would be likely to lead to higher performance and new innovations. Thus, from a broader perspective, we hope our dataset is part of the “virtuous cycle” of greater data access leading to algorithmic innovation—in turn leading to new datasets being created.

## ACKNOWLEDGMENTS

Our grateful acknowledgment goes to the students who coded and transcribed these data, and to the individuals who were filmed in the recording sessions. Special thanks to Julia Iannucci for work managing elements of this dataset.

## FUNDING INFORMATION

JS, National Institutes of Health (http://dx.doi.org/10.13039/100000002), Award ID: R03 #HD09147.

## AUTHOR CONTRIBUTIONS

JS: Conceptualization: Supporting; Methodology: Equal; Data Collection: Equal; Funding: Lead; Supervision: Lead; Writing - Original Draft: Equal; Writing - Review & Editing: Lead. MM: Analysis and Coding: Lead; Data Collection: Supporting; Supervision: Supporting; Writing - Original Draft: Equal; Writing - Review & Editing: Equal. AP: Conceptualization: Equal; Data Collection: Equal; Methodology: Equal; Supervision: Supporting; Writing - Original Draft: Equal; Writing - Review & Editing: Supporting. EW: Conceptualization: Supporting; Data Collection: Equal; Methodology: Supporting; Supervision: Supporting; Visualization: Supporting; Writing - Original Draft: Equal; Writing - Review & Editing: Supporting. MCF: Conceptualization: Equal; Data Collection: Supporting; Funding: Supporting; Methodology: Equal; Supervision: Supporting; Writing - Original Draft: Equal; Writing - Review & Editing: Supporting.
